# Introducing Temporal Theory to the Field of Sport Psychology: Toward a Conceptual Model of Time Perspectives in Athletes’ Functioning

**DOI:** 10.3389/fpsyg.2018.02772

**Published:** 2019-01-11

**Authors:** Maciej Stolarski, Wojciech Waleriańczyk, Dominika Pruszczak

**Affiliations:** Faculty of Psychology, University of Warsaw, Warsaw, Poland

**Keywords:** time perspective, motivation, mood, emotion, sport performance, athlete engagement, performance appraisal

## Abstract

Time perspective theory provides a robust conceptual framework for analyzing human behavior in the context of time. So far, the concept has been studied and applied in multiple life domains, such as education, health, social relationships, environmental behavior, or financial behavior; however, its explanatory potential has been completely neglected within the domain of sport. In the present paper we provide a deepened theoretical analysis of the potential role of temporal framing of human experience for sport-related attitudes, emotions, and athletic performance. We propose a conceptual model in which time perspectives influence psychological functioning and performance of athletes via three major mechanisms: (1) magnitude and persistence of sport motivation and resulting athlete engagement, (2) regulation of affective states during sport performance, and (3) appraisal of one’s performance and coping with resulting emotions. We support the theoretical considerations based on the major assumptions of time perspective theory with research findings regarding the regulatory role of time perspectives in other life domains. We also highlight potential research paths that would allow us to empirically test the present model and determine the actual role of temporal perspectives in shaping crucial aspects of athletes’ psychological functioning, as well as levels of their sport performance.

## Introduction

Athletes, coaches, and sports activists are increasingly aware of the fundamental role of implementing psychological knowledge into training programs (cf. Gill, 2000). Practitioners have successfully applied a vast number of psychological concepts (e.g., self-determination, Ryan et al., 2009; flow, Jackson et al., 2001; or mindfulness, De Petrillo et al., 2009) that proved effective in supporting athletes’ performance and/or quality of psychological functioning in sport. Our reasoning follows this approach, seeking to answer the question of whether, in light of the current state of the art in the field, the concept of temporal perspectives (cf. Zimbardo and Boyd, 2008) could serve as a valuable tool in sport psychology.

In the present article, we aim to provide an in-depth conceptual analysis of the role that individual differences in time perspective (TP) might play in the sport domain. Taking into account the present state of research on this issue (i.e., lack of published studies on TP in the sport context), fulfilling this apparent gap in contemporary knowledge seems to be one of the priority tasks for both TP researchers and sport psychologists. TP Theory (TPT), developed by Zimbardo and Boyd (1999, 2008), with some extensions recently provided by Stolarski et al. (2018), remains our major conceptual point of reference in the present considerations, along with Self-Determination Theory (SDT; Deci and Ryan, 2000), which remains our framework in defining and understanding motivational processes, and the Cognitive-Motivational-Relational theory of emotion (CMR, Lazarus, 1991, 2000b), which we use as a basis for discussing emotion and coping phenomena.

At this point it is crucial to define the major sport-related concepts used in the present considerations. First, following WHO (2018), *physical activity* is defined as “any bodily movement produced by skeletal muscles that requires energy expenditure.” Second, we treat *exercise* as a planned and structured form of physical activity, usually repetitive, that aims to improve or maintain physical fitness. Furthermore, we understand *sport* as all possible forms of competitive physical activity, either individual or team. While taking part in such an activity, an individual uses physical ability and skills, typically providing enjoyment to him/herself, and in certain cases it also provides entertainment for spectators.

For the purposes of the present paper, it also seems essential to properly understand the similarities and differences between exercise psychology, sport psychology and performance psychology. Although it is difficult to make a sharp and ultimate distinction between these areas (see Apa Division 47 Practice Committee, 2018), following the broadly accepted narrative we treat exercise psychology as a discipline focusing on origins and psychological consequences of undertaking physical activity. Performance psychology, on the other hand, can be viewed as focusing on explaining, predicting and optimizing of the performance-oriented activities (Nitsch and Hackfort, 2016) (for a more in-depth overview see Raab et al., 2015). Finally sport psychology is a term related to the study of how psychological factors may influence sport performance, and, on the other hand, how taking part in sport activity impacts psychological outcomes, focusing mainly on competing athletes (both amateur and professional) (see Weinberg and Gould, 2010). In the present paper, unlike the authors investigating the role of temporal framing in the context of undertaking exercise we focus on the potential role of TPs in sport performance, thus our considerations belong mainly to the fields of performance psychology and sport psychology, but not necessarily exercise psychology.

Time perspective was initially defined as “the often non-conscious process whereby the continual flows of personal and social experiences are assigned to temporal categories, or time frames, that help to give order, coherence, and meaning to those events” (Zimbardo and Boyd, 1999, p. 1271). Individuals constantly switch their attention between time horizons (the past, the present, and the future), in response to situational forces, as well as internal states, habits, and personal motives (Stolarski et al., 2018). This process is possible thanks to the uniquely human ability to perform mental time travel (Suddendorf and Corballis, 2007), and remains a core part of the stream of consciousness. Despite TP’s dynamic nature, through processes of learning and cultural influences (and, plausibly, some temperamentally determined tendencies—see Stolarski and Cyniak-Cieciura, 2016), some specific *biases* (‘temporal attractors’) are being naturally developed. As a consequence, we may observe relatively stable individual differences in these ‘temporal framings’ that lead to the forming of one’s characteristic TP profile.

Stolarski et al. (2018) have recently distinguished between *trait TPs*, i.e., “stable (...) tendency to remain chronically oriented and manifest stable attitudes toward one or another of the three time horizons: the past, the present, or the future” and *state TP*, i.e., “momentary focus on an attitude toward a time horizon (the past, the present, or the future) in a given situation” (p. 613). In the present paper, we refer to both these aspects of TP, analyzing their role in particular aspects of sport activity.

Zimbardo and Boyd (1999, 2008) empirically distinguished five basic TP dimensions that can be measured using the Zimbardo Time Perspective Inventory (ZTPI): (1) Past-Negative, (2) Past-Positive, (3) Present-Hedonistic, (4) Present-Fatalistic, and (5) Future. Their approach has been consequently developed by researchers representing various countries and cultures (see Stolarski et al., 2015a). Stolarski et al. (2018) have recently summarized these efforts and proposed a novel canonical version of the model, broadening it with additional dimensions, including (6) Future-Negative, reflecting future anxiety and worry, and (7) Present-Eudaimonic, a positive form of mindful present focus, characterized by increased awareness of the present moment. Moreover, in the revised version of TP theory, the Future dimension has been replaced with Future-Positive TP. In the present paper we refer to this seven-factor TP universe.

Another fundamental concept from within the TPT is *balanced TP* (BTP; Zimbardo and Boyd, 1999). Zimbardo and Boyd (1999) defined BTP as “the mental ability to switch effectively among TPs depending on task features, situational considerations, and personal resources, rather than being biased toward a specific TP that is not adaptive across situations” (p. 1285). BTP proved to be strongly associated with a variety of well-being indicators (Boniwell et al., 2010; Zhang et al., 2013), predicting as much as 40% of their variance. These effects remains robust even after controlling for personality features (Stolarski, 2016).

BTP can be therefore treated as a vital regulatory mechanism, allowing for effective self-regulation of various affect-related outcomes. Individuals with highly balanced temporal perspectives experience more positive moods (Stolarski et al., 2014), lower stress and anxiety (Papastamatelou et al., 2015), and lesser PTSD after traumatic experiences (Stolarski and Cyniak-Cieciura, 2016). While performing a demanding cognitive task, they experience higher task engagement, lower worry, and lesser distress, which in turn results in higher levels of performance (Zajenkowski et al., 2016). They also feel time in a different way, experiencing slower passage of time, lower time pressure, lower boredom, and less routine (Wittmann et al., 2015). This increasing body of empirical results provides a solid ground for the prediction that temporal balance may also play a vital role in the context of sport where effective regulation of emotions and motivations often remains a key to high-level performance (see, e.g., Beedie et al., 2000; Wagstaff, 2014).

Beyond the profile approach to analyzing temporal perspectives, each of the dimensions distinguished in the theory may have specific effects for particular features of sport activity and performance. It seems impossible to present the great number of detailed hypotheses that could be derived from TP theory with respect to the area of functioning in sport-related context. Below we provide an overview of major conclusions resulting from an analysis of the potential role of TP in the area of sport, and propose a conceptual model illustrating the complex interplay between temporal dimensions and various features of athlete’s functioning.

## Time Perspectives in Sport: Toward a Conceptual Model of Influences

The prepotent role of TPs has been demonstrated for a variety of affective, cognitive, behavioral, and social outcomes (see Stolarski et al., 2018, for a review). Paradoxically, most people remain completely unaware of this influence (Zimbardo and Boyd, 2008). It seems that the latter claim is true also for sport psychologists: despite the clear theoretical prerequisites allowing one to presume that the influence of TP in the domain of sport could be even more pronounced than in other domains, the role of temporal dimensions in the sport context remains an uncharted territory. Some studies, however, investigated the role of temporal dimensions in the broadly understood domain of physical activity.

Shores and Scott (2007) showed that individuals with dominant Future TP displayed the greatest desire to seek physical benefits from recreation, followed by those representing Past-Positive, and Present-Hedonistic clusters. Participants representing Past-Negative and Present-Fatalistic clusters proved equally unlikely to desire physical fitness benefits during recreation. García and Ruiz (2015) followed this line of research, confirming that Future-oriented individuals tend to seek benefits in sport activity. They also showed that present-fatalists engage in sports less frequently, whereas an opposite effect was found for individuals with balanced (vs. unbalanced) TP profile.

In a study by Adams and Nettle (2009), various indicators of future orientation predicted frequency of vigorous physical activity. Guthrie et al. (2013) obtained similar results, showing that the Future subscale of the ZTPI was predictive of more frequent exercise, even after adjustment for age and education level. Henson et al. (2006) reported positive effects of both Future and Present-Hedonistic on exercise. Analogical effects of these two TP dimensions on exercise frequency were reported by Daugherty and Brase (2010). In their study the associations were significant even after controlling for personality traits. Hall and Fong (2003) designed an intervention to promote future-oriented thinking in decisions regarding physical fitness by encouraging participants to consider the future consequences of present actions. Results from a 10-week follow-up indicated that participants in the future TP condition reported increased levels of physical activity compared to the remaining groups.

The above review of previous research shows that so far researchers have focused solely on the role of TPs in predicting everyday exercise or leisure-time physical activity. None of these studies attempted to investigate individual differences in temporal perspectives in the context of actual sport performance. Based on the main assumptions of TP theory (Zimbardo and Boyd, 1999, 2008; Stolarski et al., 2018) and a growing body of data regarding robust consequences of particular TPs in various life areas, we were able to formulate a number of predictions regarding their potential role in sport-related emotions and motivations, as well as in actual sport performance (see the following section of this paper).

Results of studies based on the present conceptual model could enrich the list of individual-level predictors of sport performance (Allen and Laborde, 2014). Moreover, our conceptual considerations could become a starting point for applied research projects, aiming to introduce temporal concepts into training practice. Given that TP profile can be modified via practical interventions (e.g., Boniwell et al., 2014), the potential of this concept for interventions in sport psychology seems to be particularly high.

## The Present Conceptual Model

As highlighted above, we propose that TP is a fundamental factor, or set of factors, underpinning athletes’ psychological functioning and resulting behaviors. The role of TP may be observable on a variety of levels, including affective, motivational, and cognitive-regulation processes. Particular dimensions of TP, as well as balanced TP profile, may foster sport engagement (Guillén and Martínez-Alvarado, 2014), sport motivation (Pelletier et al., 2013), or sport confidence (Vealey, 1986), and diminish athlete burnout (Raedeke and Smith, 2001) or competitive anxiety (Martens et al., 1990), indirectly influencing performance levels and obtained results. Interestingly, TP might influence not only antecedents of sport performance, but also athletes’ reactions to their performance. Finally, as TP is, at least in part, a derivative of one’s personal and social experiences (Zimbardo and Boyd, 2008), we expect that objective performance levels and one’s subjective appraisals of the performance might result in change in athlete’s TP profile.

**FIGURE 1:**
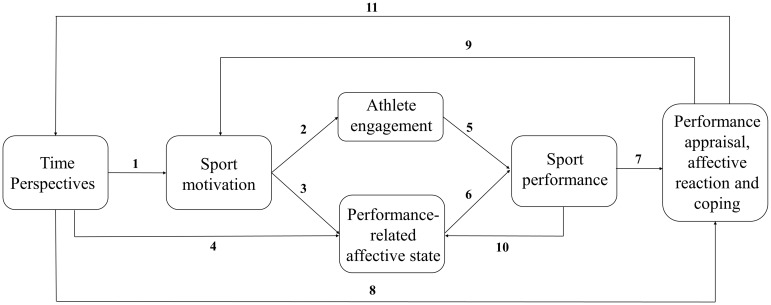
Conceptual model illustrating the interplay between time perspectives and various aspects of athletes’ functioning in sport.

Our conceptual model illustrating the hypothesized interplay between TPs and sport-related psychological states and performance is presented in Figure 1. Below we provide a detailed rationale for each pathway included in the proposed model.

### Path 1: Time Perspectives and Sport Motivation

Classic theories emphasized the critical role of future thinking in shaping motivation (Nuttin, 1964). As Simons and colleagues note, “deep future time perspective and increasing the instrumentality of one’s present behavior are associated with enhanced motivation, (...), better performance, and more intensive persistence” (Simons et al., 2004, p. 121). Although empirically confirmed mainly within the educational context, this statement appears to be universal, and motivational consequences of future focus should be analogical within the domain of sport activity, especially taking into account that Future-oriented individuals exhibit particularly high intention–behavior consistency (Van Ittersum, 2012; Villaron et al., 2017). The latter effect was demonstrated in multiple life areas, including quitting smoking (Kovač and Rise, 2007), weight control (Vinkers et al., 2013), or even participation in longitudinal research (Harber et al., 2003), and given that in sport such consistency is of particular importance (Theodorakis, 1994), analogical effects in this domain may be even more pronounced.

Classic temporal theories usually treated TP as a unidimensional construct and concentrated solely on the distinction between present and future focus (see Stolarski et al., 2018 for a review). Multidimensional approaches, such as TPT, enable much broader analyses of the regulatory functions of TP, and dimensions other than Future-Positive may also play a vital role in shaping one’s motivations and actions. For instance, it seems justified to expect negative effects of Present-Fatalistic on motivation-related outcomes, particularly due to elevated sense of helplessness and external locus of control. Although future anxiety – a core feature of Future-Negative – has some motivational properties (Carelli et al., 2011), it results in avoidant rather than approach motivations; thus in sports its consequences are probably negative, including formulating minimalistic goals or even withdrawal from demanding competition. Present-Hedonism, in turn, leads to increased baseline energetic arousal (Stolarski et al., 2014), which indicates generally elevated levels of motivation. Moreover, a striving for pleasure, characteristic for individuals situated high on this dimension, may be satisfied by the well-confirmed positive hedonic consequences of physical activity (see Berger and Motl, 2000, for a review), so that exercise-related mood enhancement may become a factor motivating such people to practice sports. At the same time, present hedonists are typically more impulsive, less persistent and may be more susceptible to dropout from a training program than their counterparts (see Harber et al., 2003; Zimbardo and Boyd, 2008); thus, effects of this dimension may strongly differ between particular aspects of motivation (e.g., intensity vs. stability) and between performance contexts (recreational vs. professional).

Further predictions in this matter could be made based on the major assumptions of SDT (Ryan and Deci, 2000), which integrates three major psychological needs necessary for the development of intrinsic motivation (IM): autonomy, competence and relatedness. For instance, induction of memories of positive past moments may foster sense of competence and relatedness, whereas reduction of Present-Fatalistic focus should result in elevated sense of autonomy: the three basic psychological needs that remain crucial for facilitating and sustaining IM and remain fundamental also within the domain of sport (Ryan et al., 2009).

Given that state-TPs can be effectively primed (Kivetz and Tyler, 2007), we also expect that transient focus on a particular time horizon should influence situational motivation, and, indirectly, enhance or diminish sport performance. Directing athletes’ attention to more distant goals and particular steps leading to realization of these goals could strengthen momentary motivation, allowing them to restrain from letting go of the training scheduled for a given day (cf. Hall and Fong, 2003), whereas reflection on positive moments from one’s past sport career could foster situational levels of their sense of competence, indirectly supporting IM.

### Path 2: Sport Motivation and Athlete Engagement

Motivation may be considered as an ‘internal state that energizes and drives action or behavior and determines its direction and persistence’ (Hagger and Chatzisarantis, 2007; p. xi). A high level of motivation is traditionally seen as a sine qua non of achieving success in sport (Orlick and Partington, 1988). It is highly unlikely that an unmotivated athlete can achieve mastery in their sport. However true the statements above, it can be argued that in the sport context, exaggerated focus is being put on quantity of motivation whereas the quality of motivation might be a focal point. Such a viewpoint is offered by SDT (Deci and Ryan, 2000) which provides a comprehensive and well-examined framework for investigating motivational processes in athletes, and has been suggested as a starting point for examination of potential antecedents for athlete engagement (AE) (Lonsdale et al., 2007).

Athlete engagement is defined as an ‘enduring, relatively stable sport experience, which refers to generalized positive affect and cognitions about one’s sport as a whole (...) a persistent, positive, cognitive-affective experience in sport that is characterized by confidence, dedication, enthusiasm, and vigor’ (Hodge et al., 2009, p. 187). Furthermore, AE can be viewed as a conceptual opposite of burnout and has a vast body of research to support that claim (Perreault et al., 2007; Hodge et al., 2008; Jowett et al., 2016).

Not only theoretical links can be found between SDT and AE, as research shows that the satisfaction of basic needs is positively associated with AE, especially in the case of competence and autonomy (Hodge et al., 2009; Jowett et al., 2016). Hodge et al. (2009) suggest that relatedness is important in the period of intense athletic development, i.e., in the earlier phases of athletes’ careers, but its significance decreases in the elite sport context.

In all, based on the abovementioned theoretical considerations and research results, it seems obvious that sport motivation naturally leads to greater engagement in training and performing. Furthermore, greater AE may lead to higher persistence in training, which in turn may affect the quality and duration of the training sessions. The result is a greater chance of developing sufficient skills to compete on a superior level. The authors of psychometric tools measuring AE (e.g., Guillén and Martínez-Alvarado, 2014) or its opposite—athlete burnout (e.g., Raedeke and Smith, 2001)—have treated motivation (or amotivation) as inherent prerequisites of these phenomena, which shows how inseparable are the constructs connected with the second pathway in the present model. Therefore, we assume, the effects of TPs on AE are mediated via general levels of sport motivation.

### Path 3: Sport Motivation and Performance-Related Affective State

Roberts (2001) states that it is impossible to point out one theory of motivation that can be described as *the* theory in a physical activity context, as different approaches can be used to explain various aspects of motivation. Detailed description of the main links between other popular theories of motivation (i.e., achievement goal theory, self-efficacy theory, the goal setting empirical approach) is outside of the scope of the present paper. Nevertheless, it is worth noting that, first, each of theories mentioned above focuses on energization, direction and regulation of behavior. Second, all of them include affect, emotions and moods, being both antecedents and consequences of motivational processes (Roberts, 2001). Since studying phenomena as fleeting as affective states is methodologically difficult, especially with definitional imprecision present in this field of study, to remedy further inaccuracy, in the present paper we will refer to affective states being the superordinate category in which moods, emotions and attitudes find space (Frijda, 1994; Gross, 2010).

Self-Determination Theory (Deci and Ryan, 2000) distinguishes intrinsic motivation (IM), extrinsic motivation (EM), and amotivation (AM). IM is defined as the “inherent propensity to actively develop skills, engage challenges, and take interest in new activities even in the absence of external prompts or reward” (Ryan and Deci, 2007, p. 2). Vallerand (2007) shows that IM can be split into three parts: *IM to know* (engaging in activities for the pleasure and satisfaction of exploring, learning, and understanding new things), *IM to accomplish* (engaging in activities for the pleasure and satisfaction of trying to exceed oneself, to create, or to accomplish something) and *IM to experience stimulation* (engaging in activities because of the stimulating sensations associated with them). EM can vary in the autonomy and integration continuum, bringing us four types: integrated regulation, identified regulation, introjected regulation and external regulation (Ryan and Deci, 2007; Vallerand, 2007). AM is described as not having intention or energy directed toward action and has a vital role in dropping out of sport (Pelletier et al., 2001) or physical activity (Ntoumanis et al., 2004).

Different types of motivation cause different outcomes in terms of psychological well-being; hence, they are the basis for various affective states (Gagné and Blanchard, 2007; Markland and Ingledew, 2007). Self-determined motivation, meaning participating in sport for more autonomous reasons, leads to more adaptive outcomes, i.e., higher levels of well-being. Involvement in sport prompted by interest and joy is related to perceived satisfaction and competence, while involvement motivated by appearance and body image is associated with anxiety and depression (Frederick and Ryan, 1993). Intrinsic motivation and identified regulation are associated with greater positive affect, enjoyment, and satisfaction whilst external regulation and AM is related to elevated anxiety and lower levels of psychological well-being (Gagné and Blanchard, 2007). Therefore, IM has indisputably beneficial effects on performance-related affective states (as intrinsic motives are markedly associated with greater psychological needs satisfaction). Although EM is usually associated with experience of being externally controlled, which leads to deleterious effects on participation-related affective states (e.g., sport dropout or burnout), it can be also experienced as self-determined and autonomous when athletes identify with and highly value the outcomes of their sport activity (*identified regulation)* or have integrated sport participation in their core values and beliefs (*integrated regulation)* (Hagger and Chatzisarantis, 2007; Markland and Ingledew, 2007). Thus, the emotional effects of EM, as a complex continuum, might change from undesirable to definitely positive along with progressive integration of extrinsic goals.

### Path 4: Time Perspectives and Performance-Related Affective States

Recent papers on TPs have highlighted the fundamental role of temporal perspectives in emotion regulation and emotional adaptation (Stolarski et al., 2014; Matthews and Stolarski, 2015). The regulatory role of TPs was demonstrated not only in a neutral context (e.g., Stolarski and Matthews, 2016), but also in task performance (Zajenkowski et al., 2016). The latter research showed that TPs’ effects on performance are mediated by task-related affective states. Emotions are crucial predictors of sport performance (Woodman et al., 2009; see also the description of Path 6 below); therefore, we expect that the regulatory role of TPs in affective processes is highly plausible in the context of sport. Hitherto, studies showed that Past-Negative and Future-Negative are robust predictors of negative affectivity (e.g., Stolarski and Matthews, 2016), Future-Positive and Present-Hedonistic predict elevated levels of energetic arousal (Stolarski et al., 2014), and Past-Positive and Balanced TP seem to underpin superior emotional regulation abilities (Stolarski et al., 2011).

It seems highly probable that these effects will replicate, or even prove more robust, in the case of athletic performance. Intrusive ruminations about past failures, characteristic of Past-Negative TP, may foster performance related stress. Similar effects, albeit related more specifically to fear of a possible loss, seem highly probable in athletes with Future-Negative bias, whereas the Present-Fatalistic perspective will plausibly result in diminished energetic arousal during competition (see Zajenkowski et al., 2016). Past-Positive, in turn, may serve as a valuable resource for building the sense of self-confidence and attenuate stress reactions through recalling past successes and positive feedback regarding one’s athletic capacity. Present-Eudaimonic should allow the athlete to engage in mindfulness-based regulation of affective states (see Hayes and Feldman, 2004).

Time perspective may influence emotional reactions to the situation of both training and competition. In the case of the former, the major mechanism responsible for this association would be overlapping with the variety of mechanisms described above for Paths 1–4, and resulting in elevated energetic arousal (Matthews et al., 1990), which is typically treated as the affective component of motivation. In the situation of competition, the regulatory role of TPs seems more complex, but it may also prove much more robust, as emotions experienced while competing remain one of the crucial factors responsible for performance level, particularly in elite athletes (see the description of Path 6 below). Adaptive temporal profiles (see Stolarski et al., 2015b) should diminish anxiety, worry and tension associated with sport performance, and foster experiences of energy and pleasure during athletic activities (cf. Matthews and Stolarski, 2015; Zajenkowski et al., 2016). The effects should be observable both in self-reported emotions, and objective, physiological outcomes (e.g., salivary cortisol).

All in all, we expect that TPs may influence performance-related emotions in three ways. First, they may influence them directly, as people varying in levels of some TP dimension also differ in baseline levels of particular affective states (e.g., habitual Future-Negative focus is associated with elevated anxiety). Second, certain strategies in temporal framing of current experience may provide effective emotion regulation strategies, allowing athletes to effectively deal with maladaptive states emerging during competition (e.g., Past-Positive-based reappraisals of the situation; see Stolarski et al., 2011, 2016; Matthews and Stolarski, 2015). Finally, TPs could influence performance-related affective states indirectly, through fostering (e.g., Future-Positive) or diminishing (e.g., Present-Fatalistic) sport motivation (pathway 1), which in turn will influence affective evaluations of sport, both at general level, and in its specific domains. For instance, taking the Future-Positive perspective should make goal-related emotions (e.g., mental representation of future success) more accessible—a resource that may be used, e.g., to overcome the famous Kilometer 30 crisis in marathon runs (Schüler and Langens, 2007).

### Path 5: Athlete Engagement and Sport Performance

It seems nearly impossible for an athlete to achieve an expert level without countless hours spent in training. As well, spending countless hours during training is difficult to imagine without an adequate amount of AE. The notion above is supported by the classical research on expertise that emphasizes the role of experience in one’s sport (Chase and Simon, 1973) and deliberate practice (Ericsson et al., 1993) in achieving mastery of the skills needed to compete at an elite level. AE can be characterized as a blend of confidence, dedication, enthusiasm, and vigor (Hodge et al., 2009), and can be viewed as a factor that may potentially improve sport performance in at least three ways.

Kristensen (2013) showed that there is a positive association between AE and time spent in training per week. Thus, in accordance with the abovementioned works (Chase and Simon, 1973; Ericsson et al., 1993), it can be assumed that athletes with a higher level of AE spend more time in training, which is a starting point for achieving an improved level of sport performance. One may also argue that with higher AE, not only an increase in quantity but also an increase in quality of training can be observed.

Secondly, AE can be seen as an adversary of burnout. This notion is supported by empirical evidence (i.e., DeFreese and Smith, 2013; Jowett et al., 2016; Martínez-Alvarado et al., 2016). Burnout, as defined by Maslach and Jackson (1984), consists of three dimensions: emotional exhaustion, depersonalization, and reduced performance accomplishment. Therefore, AE can be argued to have a beneficial effect on sport performance by impeding the negative influence of burnout.

Finally, AE has been shown to create a solid base for more frequent flow experiences (Hodge et al., 2009). The state of flow is widely considered to be a factor that supports performance in sport with both anecdotal and empirical evidence available to support this thesis (Jackson and Csikszentmihalyi, 1999; Jackson et al., 2001).

In the light of the above mechanisms, it seems justified to expect effects of TPs on performance levels mediated via sport motivation and resulting engagement in training and competition (see also Paths 1 and 3). The motivation-engagement-performance chain should theoretically depend mainly on Future-Positive and Present-Fatalistic, obviously with desirable influences of the former, and disruptive effects of the latter. Importantly, the prediction is not limited to trait-TPs only: Hall and Fong (2003) showed that a brief intervention priming future focus resulted in an increase in participants’ physical activity. Thus, situationally induced temporal focus could also foster performance via boosting motivation and persistence.

### Path 6: Performance-Related Affective State and Sport Performance

The fundamental question of whether affective states can predict performance has been asked in several studies, including different conceptualizations, definitions and, as a result, utilizing different measures. Probably the most popular approach was using the Profile of Mood States (POMS; McNair, 1971), which enables the creation of a mood profile consisting of six scales: anger, confusion, depression, fatigue, tension, and vigor. These studies find that the optimal combination of these factors, known as the “iceberg” profile, consists of low levels of anger, confusion, depression, fatigue, and tension, and a high level of vigor (Morgan, 1980). The utility of POMS in predicting performance outcome is shown clearly in the meta-analysis conducted by Beedie et al. (2000) in which moderate effects of vigor, confusion and depression, a very small effect of fatigue and equivocal effects of anger and tension on performance were shown. Furthermore, researchers observed that these “effects were larger in sports of short duration, in sports involving open skills, and where performance was judged using self-referenced criteria” (Beedie et al., 2000, p. 3). On the other hand, POMS scores were neither effective in differentiating elite and non-elite athletes nor predictive of performance when athletes differed substantially in fitness or skill level (Terry, 1995). The latter conclusion offers an explanation as to why a significant correlation between affective state and performance is not found in every study—for the link to unfold, homogeneity in factors of primary importance to the performance is needed (i.e., level, of ability, physical preparation, lack of injury or illness). Other measures, based on different conceptualizations, such as PANAS or UMACL, can present links beyond those shown above, but their detailed description remains outside the scope of the present paper (cf. Ekkekakis, 2012).

Expanding on pre-competition affective states’ role in sport performance, Hanin (2007) argued that this influence is far from being straightforward, as an adequate level of unpleasant emotions (such as anger, tension, and anxiety) may prove beneficial in performance. Furthermore, an excess of pleasant emotions may lead to losing focus, underestimating the demands of the task and generating a non-optimal level of arousal, in turn leading to imperfect performance (Hanin, 2010).

As the above evidence demonstrates that sport performance can be impacted by changes in affective states, it is vital to ask: what is the underlying mechanism of that impact? Lazarus (2000b) proposes that affective states can affect performance through their influence on motivation, physical functioning and cognitive functioning. One of the key functions of emotions is energizing behavior and channeling additional mental and physical resources toward achieving one’s goal. Furthermore, different emotions may lead to diverse ways of using that energy (e.g., fear can steer one away from an object whereas anger can lead toward an object) (Vallerand and Blanchard, 2000). Emotions can also impact the athlete’s arousal level, leading to improvement or impairment in performance depending on the complexity of the task (Jones, 2003). Finally, cognitive functioning may be affected both by emotions and changes in arousal. Parfitt et al. (1990) showed that working memory functioning can be impaired in high-arousal conditions, and Jones and Cale (1989) demonstrated that higher arousal may improve perceptuo-motor speed. Possible reduction of cognitive resources in the presence of worry has also been empirically demonstrated (Moran, 2016). Woodman and colleagues conducted experiments which showed that emotions facilitate performance if they are associated with the task demands, e.g., anger may be helpful in combative and contact sports, and hope can increase mental effort and improve reaction time as well as support higher endurance (Woodman et al., 2009). Anxiety can increase cognitive activity and information processing, which consumes working memory capacities, leaving less attention for task performance (Vast et al., 2010).

Temporal dimensions, particularly Past-Negative and Present-Fatalistic, as well as a Balanced TP profile, were shown to influence task-related affective states (worry, distress, and task engagement; see Zajenkowski et al., 2016), indirectly influencing performance in a challenging fluid intelligence test. We may thus assume that the effects of TPs on task-related moods or emotions (see Path 3) may subsequently influence performance, particularly during competition, when levels of emotional arousal are naturally higher, emotional management is particularly difficult (Landers, 1980), and adaptive emotion regulation remains the key to success (Lane et al., 2012; Wagstaff, 2014).

### Path 7: Sport Performance Appraisal

As Maehr and Nicholls (1980) suggest, success and failure are psychological states perceived by athletes determined by their interpretation of how effective their achievement striving is. In other words, a performance outcome is likely to be seen as a failure if an athlete recognizes that it indicates undesirable attributes of him- or herself (lack of ability, low effort, etc.). On the other hand, the outcome can be seen in terms of success if the desirable attributes of the self are detected (high skills or effort, etc.). From this point of view, both winning and losing are affectively involving (McAuley et al., 1983). Going further, every sport performance may also be evaluated by an athlete with one of four different appraisal patterns, based on the event’s time locus and its gain/loss potential. Namely, anticipated performance can be seen as a challenge or a threat, but most importantly: past performance may be appraised as beneficial or harmful (Lazarus, 1991, 2000b). Hanin (2007) underlines the emotion-evoking potential of those appraisals. In that regard, it is important to note that emotions perceived by an athlete are not simply generated by the performance. Their appearance is, rather, provoked by the above-described process of appraisal in which both motivational and cognitive components are contained (i.e., goals and their importance, personal beliefs, resources and environmental factors, Lazarus, 2000a,b).

People tend to assess their own performance. They do it automatically and, naturally, in a subjective manner. The outcome depends both on internal standards and peer comparisons (Horn and Hasbrook, 1987) and produces affective reactions that may by their nature have secondary effects on motivation, engagement and performance (see Paths 9–11). Despite the subjective nature of the performance appraisal, actual performance level remains a fundamental source of the appraisals and resulting emotions. These simple processes are what Path 7 actually depicts.

### Path 8: The Effects of Time Perspectives on Performance Appraisal, Affective Reactions and Coping

The role of TPs in one’s psychological reactions to their performance may be particularly robust. Let us consider a situation of underperformance in an important competition: For an athlete with high levels of Past-Negative, such a situation would be plausibly built into a negative vision of the past and the self, and result in even more negative expectations toward the future: Stolarski et al. (2014) showed in a longitudinal study that elevated levels of Past-Negative may produce a negative bias in future expectations. An athlete with generally low past focus, but characterized with high levels of Future-Positive orientation, would probably pay little attention to the result (as it already became a part of the ‘irrelevant’ past) and almost immediately turn their focus to the next career challenge. Thus, we presume that the way in which objective sport performance is interpreted by the athlete, and how important it remains for them, may partly result from individual differences in temporal framing. TP may thus constitute mental frames for perception and interpretation of one’s performance and results.

Such differences in immediate appraisals of sport performance may have vital implications for both the magnitude and persistence of resulting emotional reactions (Lazarus, 1991, 2000b). Past foci might result in athletes having more prolonged affective reactions to their own successes and failures. Consistent with that argument Ely and Mercurio (2011) showed that individuals scoring high on Past-Positive not only remember autobiographical memories better, but also recall greater emotional content of these memories and tend to relive them in the present moment. Similar effects seem probable for performance-related memories. Depending on the temporal perspective taken, cognitive representations of performance may be more vivid or pallid (Strack et al., 1985), resulting in different emotional reactions to the performance—in terms of both their quality and their persistence. It seems probable that Past-Positive athletes will experience greater and longer lasting satisfaction if they perform (subjectively) well, whereas individuals with high Past-Negative will ruminate more about their failures. Taking into account its marked links with emotional intelligence (Stolarski et al., 2011), Past-Positive may be treated as an emotion-regulation feature. Matthews and Stolarski (2015) build upon this line of reasoning and suggest that adopting Past-Positive may allow to reappraise one’s experience, even those initially treated as negative, through analyzing them from a novel, broader perspective [e.g., “I have learned a lot from this failure, and I won’t make similar mistakes in the future”; see also Zimbardo and Boyd (2008) for other examples illustrating the process of reconstructing difficult past experience].

Together, the Future-Positive and Past-Positive dimensions form a basis for a “time-expansive” attitude toward time (cf. Webster, 2011)—a broad personal temporal horizon that remains the essence of Balanced TP and allows one to go beyond “here-and-now.” Individuals characterized with such a temporal profile regulate their emotions and behaviors more effectively and report greater levels of well-being (cf. Stolarski, 2016; Stolarski et al., 2018). Such a temporal horizon also seems highly adaptive in the sport context—athletes with a more balanced TP may perceive their own successes and failures in a much broader context. Consequently, they are capable of regulating their reactions more effectively, presumably being able to overcome post-failure despondency, and transform positive, success-related euphoria into action aiming into further improve themselves (see also Paths 9 and 10).

Finally, particular TP dimensions may foster different coping strategies (cf. Endler and Parker, 1990). By their nature, Past-Negative and Future-Negative dimensions should promote emotion-oriented coping (see Matthews and Stolarski, 2015), whereas avoidance-oriented coping seem highly probable in present-oriented individuals, as both present dimensions are positively related to avoidant procrastination (Díaz-Morales et al., 2008). Future-Positive should naturally foster task-oriented coping (see Matthews and Stolarski, 2015, for a broad rationale for expecting this association).

To sum up, certain TP profiles, particularly a balanced TP, may allow athletes to (1) assess their own sport performance in a more adaptive way, and (2) deal with performance-related emotions more effectively. This, in turn, may have vital consequences for both general motivation and resulting AE, and performance-related affective states (see our discussion of Paths 9 and 10 below).

### Paths 9 and 10: Performance Appraisal, Affective Reaction and Coping Affects Sport Motivation and Performance-Related Affects

Coping can be defined as a way in which “we manage or regulate our emotions, for example, by suppressing their expression, addressing and changing the environmental or personality conditions that provoked it, or reappraising the personal significance of what has happened or is happening without changing the actual person-environment relationship” (Lazarus, 2000b, p. 235). This is a conscious process that involves cognitive and behavioral resources and mediates between appraisal, affective reaction and performance (Lidor et al., 2012).

Three basic types of coping can be defined, namely: problem-focused that involves active efforts to change the situation, emotion-focused that involves emotional regulation, and avoidance coping, which is manifested by resigning from actively participating in situations (Endler and Parker, 1990). Thus, different types of coping may lead to various affective states and, moreover, can influence motivation (Lidor et al., 2012).

Coping and affective reactions are continuously influenced by how one evaluates their performance. During and after sport performance, athletes can focus on both positive (self-empowering) and negative (self-defeating) statements that can have positive or negative influence on sports activity. Those statements result in different affective states, seen as negative (unpleasant) or positive (pleasant) as well (Lazarus, 2000b; Hanin, 2007). Those self-statements and the way athletes manage their affective states influences motivation. Ruminations also have a huge impact on sport motivation, as they often are the product of “emotional struggles” (Lazarus, 2000b, p. 249) and result in giving up when not performing well enough. Thinking about being forced and pressured by parents or coaches as well as not linking training and competition effort with outcomes can lead to AM and burnout (Gould et al., 1996).

It is important that an athlete learn how to cope with destructive self-statements that negatively influence an appraisal, coping and affective reactions, and finally sport motivation (Lazarus, 2000b). There are some strategies that can help athletes in coping, e.g., self-statement modification, imagery, Socratic dialog, corrective experiences, modeling, self-analysis, storytelling, metaphors and reframing (Jones, 2003; Vast et al., 2010).

Time perspectives may have robust effects on coping strategies and emotional regulation processes, influencing the way in which athletes react to their own performance (see our discussion of Path 8). Through this influence they may indirectly influence both resulting motivation (Path 9) and affective states experienced while performing (Path 10). Thus, the effects of TPs on these two aspects of athletes’ functioning may be not only direct (Paths 1 and 4) but also mediated via the way in which they deal with all the psychological consequences of their performance.

### Path 11: Performance Appraisal, Affective Reaction and Coping Affect Time Perspectives

How athletes evaluate their performance can affect not only their sport motivation and emotion, but also, reciprocally influence their TPs. Zimbardo and Boyd (2008) have emphasized that individual differences in TPs in large part result from social and personal experiences. Indeed, results of a study conducted on a sample of motor vehicle accident survivors showed that the magnitude of trauma exposure was associated with decreased scores on Past-Positive and Future (positive) dimensions, elevated Present-Fatalistic and greater deviation from the Balanced TP (Stolarski and Cyniak-Cieciura, 2016).

In the context of sport, one’s objective results and their subjective interpretations, social comparisons with other athletes, as well as feedback obtained from their environment (coaches, peers, parents, media, etc.), and their history of injuries, all become stored in one’s autobiographical memory, become a part of the athlete’s identity, and may influence fundamental personality features, such as self-esteem (Horn and Horn, 2007; Smith and Sparkes, 2012; O’Rourke et al., 2014). They might also—temporarily or permanently—impact individuals’ TP profile. For instance, an experience of an unfair loss may elevate Present-Fatalistic, whereas an accumulated series of losses could foster Past-Negative focus. Quite the opposite, a series of successes, but also experienced social support, may strengthen Past-Positive focus. Many other effects of sport experiences on individual TP profiles obviously also seem possible. At this point, however, we intended to emphasize that the effects of TPs on functioning in the sport domain are not simply one-way influences.

## Three Major Mechanisms Underpinning the Dynamics Presented in Our Conceptual Model

As it can be seen in the description above the hypothetical associations between TPs and various aspects of sport-related states and sport performance are probably complex and multi-level. The proposed model includes both direct and indirect effects of temporal perspectives, and feedback loops between included variables are also possible. However, the model may become much more intelligible when we approach it from a meta-level. We believe that all the dynamics included in the conceptual scheme could be brought down to three major mechanisms (or groups of mechanisms) of the influence of TPs on athlete’s psychological functioning and performance: (1) magnitude and persistence of sport motivation and resulting AE, (2) regulation of affective states during sport performance, and (3) appraisal of one’s performance and coping with resulting emotions.

The ‘motivational’ function of TP is reflected Paths 1, 2 and 5. It depicts how processes of TPs may determine levels of athletes’ motivation, indirectly influencing their engagement in training and performing, and (again indirectly) affect their performance. Additional effects of the motivating role of TP are visible in Path 3, which shows that individual motivation levels are essential for performance related affects. The second broad mechanism refers to the regulatory role of temporal framing in performance-related emotions. TPs may impact emotions and affects experienced while performing (Path 4), indirectly influencing performance levels (Path 6). The regulatory role may be reflected in both baseline, performance-related moods, as well as to emotion-regulation strategies applied while performing. Finally, the ‘performance-appraisal’ mechanism of TP influence is depicted in Path 8: taking particular TPs may lead to completely different way of assessing (Path 7) and dealing with one’s own performance. Importantly, these appraisals may further indirectly impact both the level of motivation (Path 9) and emotion experienced during subsequent performances (Path 10) or even reciprocally influence one’s habitual tendency to ‘use’ particular TPs (Path 11).

To sum up, although the present conceptual model includes a variety of interrelated constructs the mechanisms lying at its core refer to three simple, albeit fundamental aspects of athletes’ functioning. Understanding the role of TPs in these three areas may allow for the development of vital practical interventions which can be used by sport psychologists and coaches in working with athletes.

## Trait-TP vs. State-TP: Implications of The Distinction for Sport Psychology Interventions

Given that recent conceptualizations distinguish between trait-TP and state-TP it seems important to consider how each of these aspects of TP may be applied to sport psychology. As Stolarski et al. (2018) note, the effects of trait-TP on behavior are always mediated through state temporal focus. Sport psychology interventions aiming to foster one’s sport performance through enhancing or diminishing particular temporal foci may therefore be twofold. First, a desirable momentary temporal focus may be primed directly in a situation of performance (e.g., a marathoner could recall one of their past successes in a moment of crisis). Second, a systematic coaching program could be developed aiming to permanently enhance one’s trait-TP. In both these cases, a potential improvement comes from the fact that the athlete adopts a desirable state-TP while training or performing. However, in the former case the temporal focus is primed only on a single occasion, whereas in the latter the change is stable and its effects may be observable across multiple situations, both related and unrelated to the sport context. Although situational temporal primes may prove effective in some cases, to obtain a stable progress in one’s performance, a systematic change in their TP profile seems necessary. However, it seems worth noting that given that (trait) TP is often described as a *habitual tendency* (Zimbardo and Boyd, 1999, 2008), regular directing one’s attention to (a) desirable time horizon(s) using temporal primes may result in a gradual shift in their trait-TP profile.

## Toward a Verification of the Model: Directions for Empirical Research

The conceptual model presented and justified above remains the very first step to applying the TP Theory to the area of sport psychology. Obviously, the next stage would be to test the model empirically. Taking into account its complexity, the verification is hardly possible in one study, or even one research program. Below, we highlight some possible directions for researchers striving to test it in practice.

First, studies should establish TP’s nomological network in the context of sport. Such efforts have been successful in many life domains [see Appendix A in Stolarski et al. (2015a)]. Linking TPs to various constructs highlighted in the model (e.g., sport motivation, AE, sport anxiety, burnout, etc.) would provide initial information regarding the model’s validity.

Second, longitudinal studies including both TP dimensions and other variables in the previously established nomological network would allow for drawing conclusions regarding the causality of the obtained associations. As our conceptual model shows that in many cases the hypothesized effects are reciprocal, however, determining the strength of each path would allow determination of which paths are truly robust and therefore particularly relevant for potential applications in sport practice.

Third, experimental and quasi-experimental designs would be useful to test the actual role of TPs in sport performance. The former may include studying the effects of TP based coaching (Boniwell et al., 2014) or group trainings (Oyanadel et al., 2014). The latter could investigate the effects of trait TPs on various features of actual sport performance and potential mechanisms underpinning this influence (e.g., changes in affective states during competition). This research pathway would also prove truly vital for determining the legitimacy of eventual TP-based practical interventions.

Fourth, comparisons of TP profiles and their role between different disciplines or groups of sports would allow specificity/generalizability of particular paths of the proposed model. A detailed look at the consideration presented above could lead to a pertinent prediction that particular TP dimensions and particular pathways of the model may differ in their significance in qualitatively different sport disciplines. For instance, ongoing regulation of emotion (Paths 4 and 6) might be substantial in sports requiring high accuracy (e.g., archery; see Robazza et al., 2002), whereas maintaining high levels of motivation and persistence (Paths 1, 2, and 5) should prove crucial in disciplines requiring supreme endurance (e.g., distance running; Schüler and Langens, 2007).

Fifth, TP profiles may shed some new light on one of the biggest issues in contemporary psychology of physical activity, namely dropouts, especially in youth sport (Crane and Temple, 2015). We argue that high Future-Positive orientation may prove to be a protective factor, whereas Present-Fatalistic and Past-Negative perspectives can predict earlier resignation from sport participation. Interesting and somewhat surprising support for this thesis may come from a study conducted outside the sport context by Harber et al. (2003) in which Future-orientation was a significant factor that supported prolonged participation in studies.

Sixth, development of an entirely new measure of individual differences in TPs crafted particularly for the domain of sport seems to be a particularly interesting option that could make the concept more adjusted to the area and allow for conducting more ecologically valid research. Domain-specific individual difference metrics are now increasingly valued, as a growing body of data shows that human behavior may differ between various areas of functioning (Blais and Weber, 2006), and scales developed for particular life domains may be characterized with greater validity. Within the domain of sport, domain-specific measures were developed for such constructs as burnout (Raedeke and Smith, 2001) or perfectionism (Waleriańczyk and Stolarski, 2016).

Finally, taking into account certain overlap of TP dimensions with well-established personality features (e.g., Kairys and Liniauskaite, 2015), it seems important to test for TPs’ incremental variance in explaining variability in sport-related outcomes, controlling for such basic and well established predictors as Big Five personality traits (Piedmont et al., 1999), perfectionism (Waleriańczyk and Stolarski, 2016), or self-esteem (O’Rourke et al., 2014). This refers to all research pathways suggested above: including control variables will allow determination of the actual robustness of potential findings, and will allow avoidance of the charge of “selling old wine in new bottles” (e.g., Antonakis, 2004). This issue seems particularly important.

## Conclusion

In the present paper we have introduced a conceptual model that provides a theoretical framework for applying TPT (Zimbardo and Boyd, 1999; Stolarski et al., 2018) to sport psychology. We believe that the presented review of potential dynamics between TPs and sport-related outcomes, as well as the proposed outline of potential research paths, could become a valuable starting point for extensive study of the role of individual differences in temporal framing in the functioning of athletes. Such research endeavors could, in turn, provide necessary information for applying the concept to the practice of athletes, coaches and sport psychologists. Importantly, outcomes of such studies do not have to be limited to the area of sport: in the present consideration we grounded our predictions on a variety of conclusions originating from the research conducted in other areas of human functioning. *Per analogiam*, particularly given the ecological validity of studies conducted in the domain of sport is relatively high due to the access to objective indicators of performance (see also Davids, 1988), empirical and conceptual findings regarding the temporal underpinnings of athletes’ functioning could provide some novel knowledge regarding the nature of TP, leading to further extensions of TP theory. They could also become a valuable point of reference for studying the regulatory role of TP in other life domains, such as education, work, or financial behaviors.

## Limitations

Although the present article introduces a novel, potentially valuable conceptual framework that could be introduced in the area of sport performance research and practice, certain limitations need to be pointed out. First, the model remains purely conceptual and thus speculative: any empirical support for the formulated predictions comes from studies conducted in completely different areas of human functioning (e.g., education). Reliable studies carried out within the context of sport performance are necessary to provide actual support for the proposed model. Second, although some results (e.g., Oyanadel et al., 2014) suggest that individual TP profile can be modified through interventions, little is known about actual effectiveness of such efforts. Further research is necessary to develop valid intervention programs aiming to modify individuals’ TP profiles. Moreover, the amount to which TP could be changed still has to be determined: despite Zimbardo and Boyd’s (1999, 2008) claims regarding mainly environmental and cultural roots of TPs, particular dimensions distinguished in their model are vitally associated with certain personality dimensions (e.g., Future-Positive is related to conscientiousness, whereas Past-Negative – with neuroticism; see Stolarski and Matthews, 2016) that have been proved to be markedly heritable (e.g., Riemann et al., 1997). Genetic influences on the individual tendency to adopt particular temporal perspective could limit effectiveness of any intervention programs.

## Author Contributions

MS, WW, and DP developed the conceptual model. MS wrote the Introduction, parts of the detailed model description referring to time perspective theory, a part of Directions for empirical research, and Conclusion. WW wrote parts of the detailed model description referring to emotion, emotion regulation processes, sport performance, and performance appraisal, as well as a part of Directions for empirical research. DP wrote parts of the detailed model description referring to motivation, athlete engagement, coping, as well as attitude and time perspective change.

## Conflict of Interest Statement

The authors declare that the research was conducted in the absence of any commercial or financial relationships that could be construed as a potential conflict of interest.
